# Supplementary material for: Quantitative Assessment and Prognostic Associations of the Immune Landscape in Ovarian Clear Cell Carcinoma
Source: Cancers (Basel). 2021 Jul 30;13(15):3854. doi: 10.3390/cancers13153854 (PMC8345766; doi:10.3390/cancers13153854)
Supplement: Supplementary file 1 [file cancers-13-03854-s001.zip › cancers-1304093-Supplementary-Figures.pdf]

Supplementary Materials

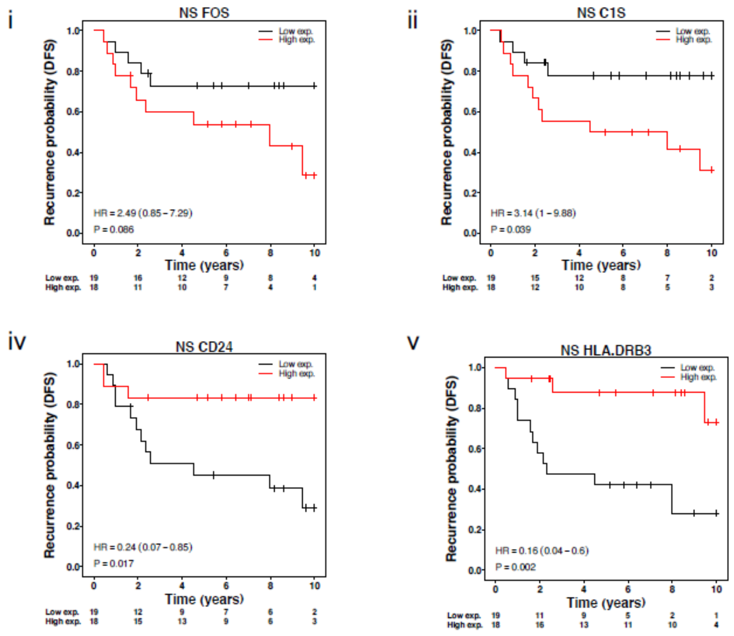

Figure S1. Prognostic genes in low and high-risk patients in the discovery cohort.

Predicted Disease Recurrence Risk in Uehara et al. OCCC (samples n = 25) [1].

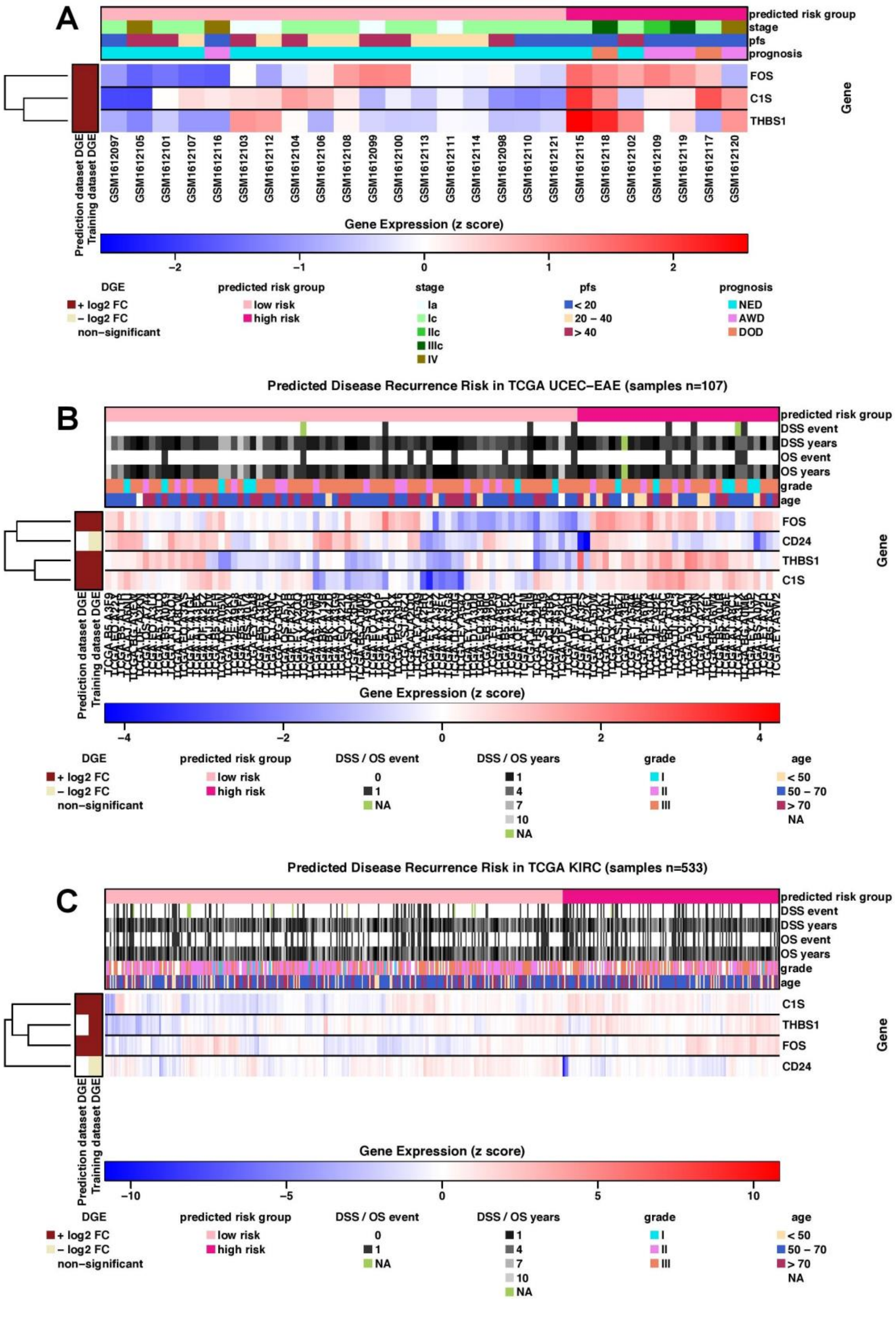

Figure S2. Predicted risk groups in the validation datasets.

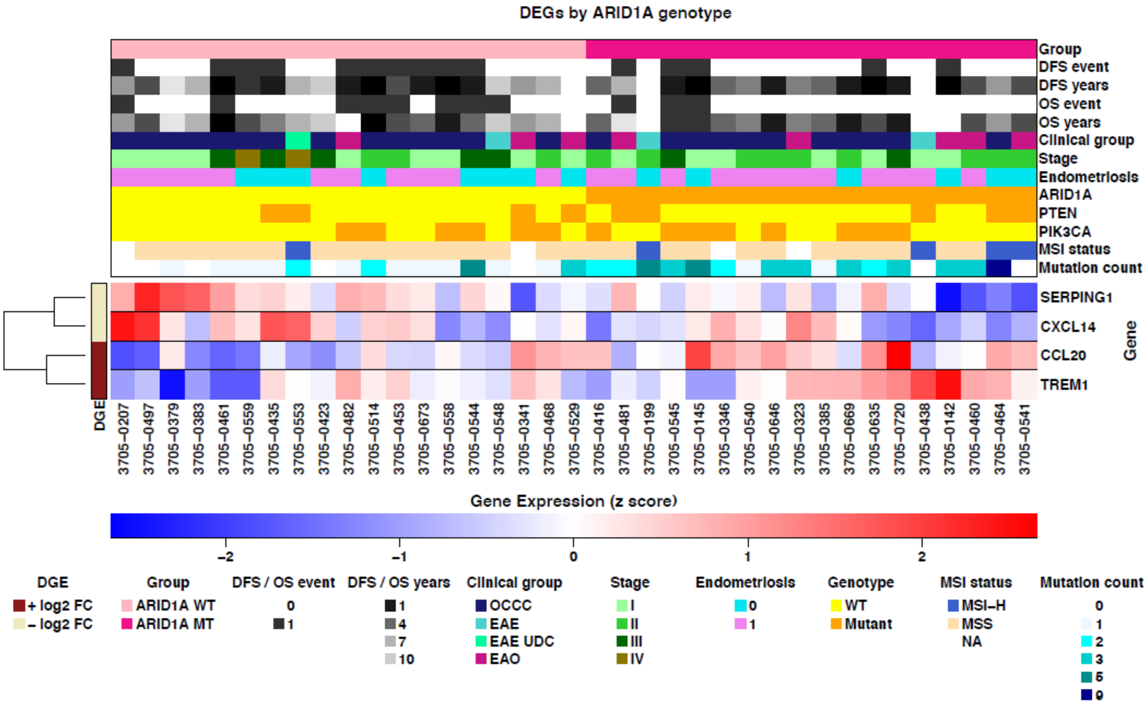

Figure S3. Differential gene expression in ARID1A mutant versus wild-type tumours.

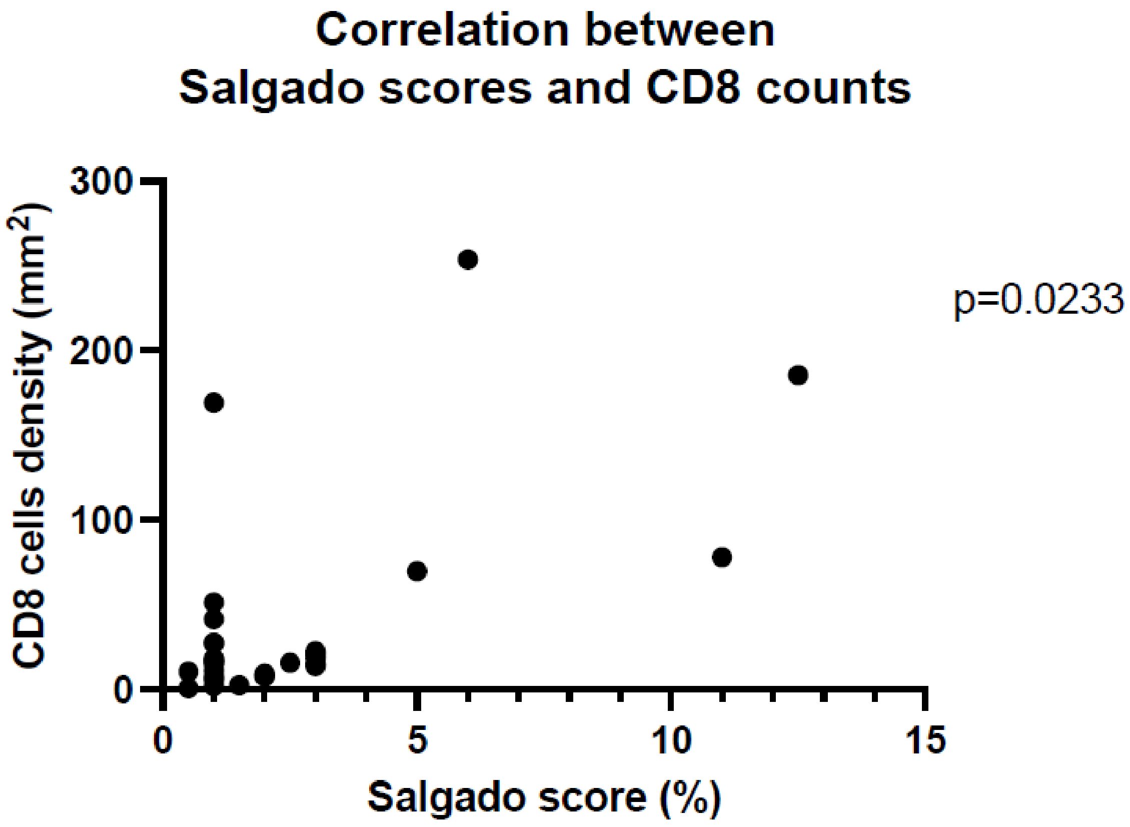

Figure S4. Correlation between Salgado scores and CD8 counts.

References

1. Uehara, Y.; Oda, K.; Ikeda, Y.; Koso, T.; Tsuji, S.; Yamamoto, S.; Asada, K.; Sone, K.; Kurikawa, R.; Makii, C.; et al. Integrated copy number and expression analysis identifies profiles of whole-arm chromosomal alterations and subgroups with favorable outcome in ovarian clear cell carcinomas. PLoS ONE 2015, 10, e0128066, doi:10.1371/journal.pone.0128066.
